# What causes amyotrophic lateral sclerosis?

**DOI:** 10.12688/f1000research.10476.1

**Published:** 2017-03-28

**Authors:** Sarah Martin, Ahmad Al Khleifat, Ammar Al-Chalabi

**Affiliations:** 1 Maurice Wohl Clinical Neuroscience Institute, King’s College, London, UK

**Keywords:** amyotrophic lateral sclerosis, neurodegenerative disease, motor neuron disease

## Abstract

Amyotrophic lateral sclerosis is a neurodegenerative disease predominantly affecting upper and lower motor neurons, resulting in progressive paralysis and death from respiratory failure within 2 to 3 years. The peak age of onset is 55 to 70 years, with a male predominance. The causes of amyotrophic lateral sclerosis are only partly known, but they include some environmental risk factors as well as several genes that have been identified as harbouring disease-associated variation. Here we review the nature, epidemiology, genetic associations, and environmental exposures associated with amyotrophic lateral sclerosis.

## Introduction

Amyotrophic lateral sclerosis (ALS) is an incurable condition, characterised by progressive degeneration of upper and lower motor neurons, resulting in paralysis and death from respiratory failure in a median of 2–3 years ^1^. Despite the poor prognosis, there is considerable variation in the survival rate, and up to 10% of people with ALS live for more than 8 years from first symptoms ^2^. Understanding what causes ALS or influences survival is crucial for the development of effective treatments.

The causes of ALS are largely unknown. Significant advances have been made in understanding the genetic and environmental components of the disease. In this report, we will explore what is known about why some people develop ALS and how the risk factors work together to cause the disease.

## Epidemiological studies of ALS

The incidence of ALS is about 2 per 100,000 person-years, and the prevalence is about 5 per 100,000 persons ^3^. Because of the low prevalence, the average primary care physician will see 1 person in their lifetime, a typical UK neurologist will diagnose about 2 people a year, while a tertiary referral centre will see more than 100 people. Despite the low incidence, however, ALS is not particularly infrequent. The lifetime risk is about 1 in 300 by the age of 85, with the risk increasing steadily, at least until about the eighth decade of life ^4, 5^. This is very similar to the risk for multiple sclerosis in the UK ^6^.

Tertiary referral centres see sufficient numbers of people that research studies will have adequate power for statistical analysis. However, there is a significant diagnostic delay in ALS, typically about a year, which seems to be independent of healthcare system and is probably related to low recognition by primary care physicians ^7^. As a result, those attending specialist centres tend to be those with a better prognosis, who are younger, and who are more motivated ^8, 9^. In contrast, population-based registers capture all cases in a defined catchment population, regardless of attendance at a specialist clinic. Such registers have provided valuable insights into the epidemiology of ALS and offer an unbiased view of the condition ^10^.

ALS can affect people at any age. The mean age of onset is 56 in clinic registers but 70 in population-based registers. In clinic registers, ALS is more frequent in men, with a male:female ratio of about 3:2, and the ratio becomes more equal with increasing age. In population registers, although the male preponderance is still seen, the ratio may be closer to 1:1, an effect that can be attributed to the greater capture of older people with ALS ^3^.

## What is ALS?

ALS presents in many different ways ( Table 1), and it has been recognised for many years that the different clinical presentations correspond with differences in survival ^11, 12^. Bulbar palsy, in which dysarthria followed by swallowing difficulty is the main presentation, is associated with the worst prognosis, and flail arm or flail leg syndrome, in which there is symmetrical, predominantly flaccid weakness of the limbs, is associated with the best prognosis ^13^. Perhaps surprisingly, statistical methods such as latent class cluster analysis can analyse the same data and identify different clinical subtypes that predict prognosis with far more discrimination than can neurologist classifications ^13^. Most cases of ALS are focal in onset and relentlessly progressive, often to contiguous regions, although there are some exceptions ^14^. The spread could be the result of a “prion-like” spread of toxic proteins through phagocytosis (consumption of cells by other cells) or possibly through a time-to-failure model ^15– 17^. Lower motor neuron failure is the main cause of weakness in ALS and can be measured non-invasively to provide data to assess cellular patterns of spread ^18^. Understanding the mechanisms of spread will aid the development of novel therapeutics and may aid models of prognosis.

**Table 1.** Clinical presentations of amyotrophic lateral sclerosis.

Classifying feature	Name of phenotype	Description
Motor neuron involvement	Amyotrophic lateral sclerosis (ALS)	Mixture of upper and lower motor neuron signs on clinical examination. Degree of certainty of diagnosis based on El Escorial criteria. May involve up to all regions.
	Primary lateral sclerosis or upper motor neuron predominant ALS	Clinical signs limited to upper motor neuron features. Generally slowly progressive but involving up to all regions. This phenotype is usually confirmed if there have been no lower motor neuron signs after 4 years.
	Progressive muscular atrophy or lower motor neuron predominant ALS	Clinical signs limited to lower motor neuron features. Slightly slower progression but can involve all regions. This phenotype is usually confirmed if there have been no upper motor neuron signs after 4 years.
Site of onset	Bulbar onset	Site of onset may be included in the description of ALS, as different disease onset patterns have different rates of progression. The two categories are bulbar and spinal.
	Spinal onset	
Disease focality	Progressive bulbar palsy	Condition involving the bulbar region and predominantly lower motor neurons. May progress to other regions.
	Pseudobulbar palsy	Condition involving the bulbar region and predominantly upper motor neurons. May progress to other regions.
	Flail arm	Predominantly lower motor neuron proximal symmetrical involvement in the upper limbs. Some upper motor neuron signs may be seen in the lower limbs.
	Flail leg	Lower motor neuron distal symmetrical involvement restricted to the lower limbs. May affect one side only.
Cognitive involvement	ALS with cognitive impairment	ALS with some cognitive involvement below the threshold criteria for frontotemporal dementia.
	ALS with frontotemporal dementia (ALS-FTD)	ALS with frank frontotemporal dementia.

The diagnosis of ALS is clinical, with the support of electrophysiological studies and the exclusion of mimics. In some cases, early diagnosis can be challenging, particularly if weakness is confined to one region for some time or is confined to a subset of motor neurons (upper only or lower only). A sensitive set of electrodiagnostic criteria, the Awaji criteria, can be particularly useful in the early diagnosis of people with bulbar onset disease, which is important because of the need for sooner gastrostomy when swallowing is affected early ^19– 22^.

ALS is classified for research purposes by the El Escorial criteria and their revisions, which improve homogeneity in recruitment for clinical trials and other clinical studies ^23– 26^. ALS progression is measured functionally using the ALS Functional Rating Scale – Revised, which uses 12 questions scored between 0 (no function) and 4 (full function) to generate a summary score ^27^. The scale is widely used but has some limitations, since the subscores correlate more accurately with progression in different clinical subtypes ^28^.

**Figure 1.**
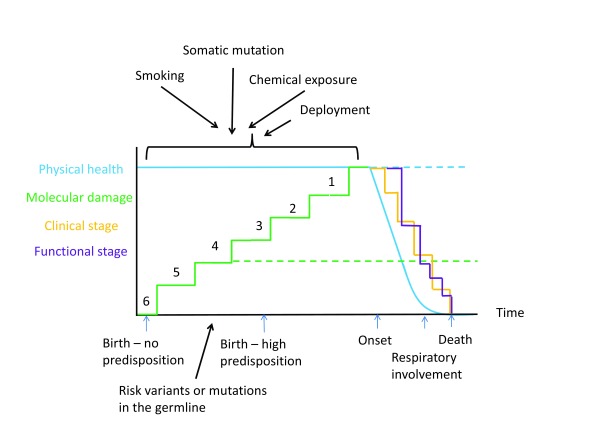
The time course of amyotrophic lateral sclerosis (ALS). Time is represented along the x-axis; physical health and molecular damage are represented along the y-axis. With time, molecular damage increases in a step-wise way until it reaches a threshold, at which point physical health declines, representing disease onset. People with a family history of ALS may have a large genetic predisposition to ALS and so need fewer steps to reach the level of molecular damage that causes disease, corresponding to a younger age of onset. Lack of exposure to sufficient risk factors means that the disease does not manifest, even if a genetic cause is present, explaining reduced penetrance. There is not a 1:1 mapping of risk factors and steps, as the steps represent molecular hits that lead to cellular damage rather than actual exposures. Once physical symptoms have started, progression shows a log-linear decline until the onset of respiratory symptoms, where decline is exponential. Clinical and functional involvement can be measured by the King’s clinical staging and Milano-Torino staging (MiToS) systems. A dotted line represents the hypothetical trajectory in an unaffected individual. Black arrows represent genetic and environmental risk factors. Numbers indicate remaining molecular hits until disease onset.

Disease staging allows a simple description of the extent of physical or functional involvement in an affected person and guides management. Such systems have been in widespread use in cancer for years. In ALS, two recent staging systems have been proposed: King’s clinical staging and Milano-Torino staging (MiToS) ^29, 30^. The King’s system is similar to cancer staging in that the clinical spread of disease is used to infer the extent of disease progression. Spread is defined as involvement producing signs or symptoms in the El Escorial domains (1 domain is stage 1, 2 domains is stage 2, and 3 domains is stage 3), with respiratory or nutritional failure characterising stage 4. The ALS functional rating scale can be used to estimate the King’s stage with 92% correlation ^31^. MiToS uses the ALS functional rating scale subscores to define functional stage ^29^. Each system has benefits in describing ALS stage succinctly. The two disease staging systems are complementary ^32^. King’s staging summarises the clinical or anatomical spread of disease. Mapping disease progression to clinical stage rather than survival could be used as a secondary endpoint in clinical trials, which would shorten trial durations and provide meaningful information on which stage of the disease is prolonged by an effective therapy ^33^. MiToS summarises the functional burden of disease. It would therefore be useful in showing a functional benefit in clinical trials. Comparison of the systems shows that functional stage lags behind clinical stage, reflecting the functional reserve available in an affected limb, and it has been proposed that a combined stage is used, as is standard in cancer, along the lines of K3M2, which would mean King’s stage 3, MiToS stage 2 ^32, 34^ ( Figure 1).

It is now recognised that ALS involves non-motor systems ^35^. Between 30 and 50% of people have cognitive impairment detectable on formal testing, resulting from involvement of the frontotemporal circuits ^36, 37^. Frank frontotemporal dementia occurs in about 5%, and in some families, people may have ALS, frontotemporal dementia, or both ^36, 38, 39^. The clinical impact of frontotemporal impairment in ALS is now more easily recognised because of recent advances in the tools available to detect it, such as the Edinburgh cognitive assessment score (ECAS) ^40, 41^. Other neurodegenerative diseases have also been linked to ALS, including spinocerebellar ataxia, in which case studies have reported the co-occurrence of ALS and cerebellar degeneration ^42^. Schizophrenia may be more frequent in families with ALS, and there may also be an increased frequency of multiple sclerosis ^43, 44^. In many of these cases, genetic factors are responsible for some of the risk. For example, pathological expansion of a repeat sequence in the *C9orf72* gene is associated with ALS, frontotemporal dementia, or both, and the same mutation may increase the risk of schizophrenia, Parkinson’s disease, and multiple sclerosis ^45^. Expansion of a repeat sequence in the *ATXN2* gene is associated with ALS or, if more than 30 repeats are involved, with spinocerebellar ataxia ^46^. Autonomic, skin, and eye movement changes are also seen. Thus, ALS is a neurodegenerative disease in which the brunt falls on the motor system, but, as for many other neurodegenerations, the clinical syndrome is also dispersed through other anatomical and physiological systems.

## Understanding prognostic factors in ALS

Respiratory impairment is usually an end-stage event in ALS. Despite this, because respiratory function is difficult to measure reliably with non-invasive methods, measurement of respiratory function is generally used as a guide to the use of respiratory support rather than prognostication ^47^. There have been many attempts at prognostic modelling, using either clinical features alone or biological markers such as albumin, creatinine, or neurofilament levels ^48, 49^. Most studies find that longer survival is associated with younger age at symptom onset, presentation with limb dysfunction rather than swallowing or speech disturbance, and specific forms of ALS such as symmetrical patterns (e.g. flail arm syndrome) or upper motor neuron predominant forms ^50^. Conversely, cognitive impairment comprising executive dysfunction, rapid weight loss, and respiratory involvement at first examination, although not necessarily respiratory onset, predict a poor prognosis ^51– 58^. The best predictor of slow progression, however, appears to be a long interval between symptom onset and diagnosis, probably because this reflects the rate of disease progression overall ^59^. Genetic variations have been associated with survival duration, with the best studied being variation in the *UNC13A* gene ^60, 61^. Variation in the *CAMTA1* gene has also been associated with survival ^62^. Furthermore, some risk genes harbour variants that are themselves predictors of prognosis. For example, the p.Asp91Ala variation of the *SOD1* gene is associated with very slow progression ^63, 64^, while the p.Ala5Val variant is associated with aggressive disease ^65^. Statistical models can be used to provide clinically useful information for patients, the strongest message being that survival is extremely unreliably predicted in individuals, even though patterns can be seen in the data ^54, 57, 66– 68^.

## Genetics and ALS

There are now more than 25 genes in which an association with ALS has been replicated, with the rate of gene discovery doubling every 4 years ( http://alsod.iop.kcl.ac.uk) ^69^. In up to 10% of people, there is a family history of ALS in a first-degree relative, but detailed genealogical studies extending to more distant relatives and including related diagnoses suggest that more than 20% have a relevant family history. The genes responsible for familial ALS have now been identified for about 70% of all cases, but there is a significant genetic component, even in those without a family history. Twin studies suggest the heritability is about 60%, and nearly every familial ALS gene has also been implicated in apparently sporadic ALS ^70, 71^. Furthermore, statistical analysis shows that the distinction between familial and sporadic ALS is not clear-cut, and large-scale genome-wide association studies (GWAS) show that the genetic architecture of sporadic ALS is one in which rare variation, more usually associated with familial disease, is disproportionately important ^72, 73^.

The most recent GWAS of ALS identified four new associations, three of which were successfully replicated ^73^. An interesting feature of the study was that even though this was a study of people with apparently sporadic ALS, there were associations in genes previously identified from family-based studies – *C9orf72*, *TBK1*, and *NEK1* – further supporting the notion that familial and sporadic ALS are not mutually exclusive categories but rather a spectrum ^74– 76^. These three genes all harbour variants that are moderately penetrant. In other words, carrying a disease-associated variant does not mean ALS will inevitably follow. Current thinking is that common diseases are the consequence of the additive effects of small increases in risk from multiple common variations (polygenic), and rare diseases are the consequence of single gene variants that are themselves rare but have a large effect on the probability of disease (monogenic). For example, height and schizophrenia are polygenic traits, while Huntington’s disease and Kennedy’s disease are monogenic diseases. ALS sits somewhere between these two extremes, with a lifetime prevalence that is far greater than is typical for a monogenic disease but far less than a common disease, and it is perhaps, therefore, to be expected that its genetic architecture also seems to sit somewhere between polygenic effects and monogenic high-penetrance disease.

There are three genes that have had a major impact on our understanding of ALS. ALS-linked dominant mutations in the superoxide dismutase gene *SOD1* were first identified in 1993, and since then mutations have been found in every exon of the gene ^77^. The SOD1 protein is a free radical scavenger, and loss of function, increasing free radical damage in cells, is a logical hypothesis to consider. However, several well-characterised *SOD1* variants do not lead to a reduction in dismutase activity, and the evidence instead supports a toxic gain of function ^78^. Transgenic *SOD1* mice develop a motor neuron degeneration and have been used to model the disease for treatment development ^79^. The second important ALS gene is *TARDBP*, which codes for TDP-43, a protein regulating RNA expression and the major component of intracellular inclusions in ALS. The discovery of ALS-linked mutations in this gene was the first of many showing RNA processing defects to be important in ALS pathogenesis and, importantly, showed that the TDP-43 inclusions were not simply a passive marker of neuronal death but a crucial part of the disease pathway ^80– 82^. The third important genetic finding in ALS was of linkage ^83, 84^ and then association ^85– 87^ of a locus on chromosome 9, which led researchers to the identification of a massive expansion of a hexanucleotide repeat in intron 1 of the *C9orf72* gene ^88, 89^. This is the most frequent cause of ALS, being responsible for about 30% of familial and up to 10% of sporadic cases.

The focus of genetic research in ALS in the immediate future is therefore on rare variation. This is best discovered through high-throughput sequencing, and this technique has already identified several familial ALS genes. The major challenge facing researchers is how to interpret the findings, since the identification of a rare variant in an ALS gene is not in itself strong evidence of relevance in that individual, and over-representation of rare variation in cases over controls in a particular gene does not provide sufficient information for genetic counselling on a specific variant ^90^. The amount of heritability explained by genetic information captured on genome-wide microarrays is about 12%, implying that the remainder is in rare variants and other types of genetic variation such as copy number variation, microsatellite repeats, post-transcriptional RNA editing, and epigenetic changes ^91^. These are likely to be the next targets of ALS genetics research and are reliant on international research consortia. Project MinE is one such global collaboration that aims to analyse DNA from at least 15,000 people with ALS and 7,500 controls ( https://www.projectmine.com/).

## Environmental risk factors

In contrast to genetics, environmental risk factors for ALS have been more difficult to identify. Such studies are expensive to perform but difficult to fund and are heavily reliant on recall ^92^. As a result, they are susceptible to bias. Furthermore, unlike genome-wide analyses, in which a hypothesis-generating approach can be taken, it is not straightforward to assay all possible environmental factors, and so a selected subset of assumed risk factors is tested. Smoking has been associated with increased risk of ALS in some studies and may hold a higher risk in some subgroups ^93^. Occupation, particularly military service with deployment, has been associated with risk of ALS, but the evidence mainly comes from the US, where there are large military datasets ^94^. Physical activity is another widely studied risk factor, partly because of a number of high-profile sports players who have had ALS and because of people with ALS having a low BMI on presentation and higher levels of leisure sports participation ^95^. It is not clear whether having higher levels of physical activity raises the risk of ALS and, if it does, whether it is the activity itself or being genetically predisposed to high sporting prowess that is the mechanism ^96^. Similarly, electric shock is not a risk factor in some analyses but is in others ^97, 98^. There is mixed evidence for the involvement of chemicals, such as heavy metals, ambient aromatic hydrocarbons, pesticides, and cyanotoxins ^99– 103^. Trauma, including head injury, also appears to be a risk factor in meta-analysis ^104^.

## Inflammation and ALS

Evidence of an immuno-inflammatory component in ALS pathogenesis is compelling ^105, 106^. A pathological hallmark of the neuroinflammation is prominent microglia activation at involved sites. T-regulatory lymphocytes (Tregs) are important immunomodulatory cells that regulate the balance between activation and suppression of the immune response and control the microglia in the central nervous system: specifically, pushing them towards a state in which remodeling and repair activities are activated. Defects in Treg levels or function have been found in ALS patients, becoming more frequent as the disease progresses. Treg levels are inversely correlated with disease severity, so that lower levels are seen in more severe disease, and survival is worse in those with Treg defects ^105– 109^. Studies are now underway to explore immune therapies that might improve Treg function and therefore improve survival.

## Retroviruses and ALS

Poliovirus and other enteroviruses can cause a post-infectious myelitis with subsequent paralysis, and HIV infection can result in an ALS-like syndrome. Studies of serum and cerebrospinal fluid from ALS patients suggested that an activated endogenous retrovirus was associated with ALS ^110^. Recently, the sequence has been identified as possibly HERV-K, an endogenous retrovirus that exists as an open reading frame in the human genome ^111^. In mice, the *env* protein component of HERV-K is toxic to motor neurons. There is no evidence that HERV-K is causative of the disease in humans, but studies are now underway to explore if antiretrovirals might slow progression and improve survival in ALS.

## Conclusion

The apparently homogeneous phenotype of predominantly motor degeneration that is ALS can result from many different causes: genetic, epigenetic, environmental, and internal. Thus, many different pathways converge on the final outcome of upper and lower motor neuron death. Careful analysis of incidence data in European population registers shows that, on average, each pathway comprises six molecular steps ^112^ (see Figure 1). The model explains many otherwise enigmatic features of ALS, such as the increasing risk with age, genetic pleiotropy (the same gene variation can result in different diseases), age-dependent penetrance of disease genes, the difficulty in identifying a single environmental cause, the observation that ALS appears to start in one region and spread, and that it is specific to motor neurons but can affect other cell types. The next challenge is to understand the extent to which the pathways overlap and therefore might be amenable to a common treatment strategy. Although ALS remains a uniformly fatal diagnosis, accelerating advances in our understanding bring the hope that an effective treatment can be found for this devastating disease.
